# Anti-malarial prescribing practices in Sudan eight years after introduction of artemisinin-based combination therapies and implications for development of drug resistance

**DOI:** 10.1186/s40360-015-0002-4

**Published:** 2015-03-26

**Authors:** Abeer Abuzeid Atta Elmannan, Khalid Abdelmutalab Elmardi, Yassir Ali Idris, Jonathan M Spector, Nahid Abdelgadir Ali, Elfatih Mohamed Malik

**Affiliations:** Al Neelain University, Steen Street, P.O. Box 7294, Code: 11123 Khartoum, Sudan; Federal Ministry of Health, Khartoum, Sudan; Gezira State Ministry of Health, Wad Medani, Sudan; Harvard School of Public Health, 677 Huntington Avenue, Boston, MA 02115 USA

**Keywords:** Malaria, Artemisinin-based combination therapy, Drug resistance, Sudan, Sub-Saharan Africa

## Abstract

**Background:**

The World Health Organization (WHO) recommends artemisinin-based combination therapies (ACTs) as first-line treatment for uncomplicated malaria. Sudan revised its malaria treatment policy accordingly in 2004. However, eight years after ACTs were introduced in Sudan the patterns of ACT prescribing practices among health care providers remain unclear. We systematically analyzed use of ACTs in a large number of primary health facilities and we discuss the public health implications of our findings.

**Methods:**

This cross-sectional study was based on WHO’s guidance for investigating drug use in health facilities. Data were collected from 40 randomly selected primary health centers in five localities in Gezira State, Sudan. The primary outcome of the study was the proportion of patients who were adequately managed according to Sudan’s recommended malaria treatment guidelines. Twelve drug-use indicators were used to assess key ACT prescribing practices.

**Results:**

One thousand and two hundred patients diagnosed with uncomplicated malaria were recruited into the study. ACT was prescribed for 88.6%patients and artemether injections were (incorrectly) prescribed in 9.5% of cases. Only 40.9% of patients in the study were correctly diagnosed and 26.9% were adequately managed according to the nationally recommended treatment guidelines. Incorrect prescribing activities included failure to use generic medicine names (88.2%), incorrect dosage (27.7%), and unexplained antibiotic co-prescription (24.2%). Dispensing practices were also poor, with labeling practices inadequate (97.1%) and insufficient information given to patients about their prescribed treatment (50.5%).

**Conclusion:**

Irrational malaria treatment practices are common in Sudan. This has important public health implications since failure to adhere to nationally recommended guidelines could play a role in the future development of drug resistance. As such, identifying ways to improve the anti-malarial prescribing practices of heath workers in Sudan may be a priority.

## Background

The World Health Organization (WHO) recommends artemisinin-based combination therapies (ACTs) as first-line treatment for uncomplicated malaria [1,2]. ACTs consist of two anti-malarial compounds: an artemisinin derivative, which induces rapid reduction of parasite load in blood over a period of days, and a partner drug, which eradicates remaining parasites [3].

Recently, artemisinin resistance has been observed in four Southeast Asian countries (Cambodia, Myanmar, Thailand, and Viet Nam) [4-6]. This has been attributed to factors including irrational prescribing practices, poor patient compliance with prescribed regimens, improper use of artemisinin monotherapies, and inadequate access to quality assured forms of the drug [7-9]. Fortunately, ACTs remain effective as long as resistance to the partner drug has not developed [10]. But while resistance to ACTs has not yet been observed, concern exists that poor treatment practices may promote ACT resistance in the future, a situation similar to the global spread of chloroquine resistance that has occured [11].

In 2011, WHO encouraged the scale-up of interventions to protect the efficacy of ACTs, which was supported by the release of the Global Plan for Artemisinin Resistance Containment [8]. Currently, WHO recommends five forms of ACTs: artemether-lumefantrine (AL), artesunate-sulfadoxine/pyrimethamine (ASP), artesunate-amodiaquine (ASAQ), artesunate-mefloquine (ASMQ), and dihydroartemisinin-piperaquine (DHAPQ) [12]. Malaria-endemic countries in Sub-Saharan Africa have adopted several of these different formulations of ACTs in their national strategies for malaria control and elimination [13].

In 2004 Sudan revised its Malaria treatment policy in favor of use of ACTs. The nationally recommended first- and second-line treatments are ASP and AL, respectively [14,15], both of which are provided free of charge at primary health care facilities in Sudan. According to national treatment guidelines in Sudan, peripheral blood smears should be obtained on febrile patients that are suspected of having malaria in order to confirm the diagnosis before treatment (presumptive diagnosis of malaria is no longer accepted for prescribing treatment, except in the increasingly rare event that no laboratory facility or rapid diagnostic testing capability are available). However, some data suggest that the malaria guidelines in Sudan are far from universally adhered to. A cluster-sample survey conducted in 15 states in Sudan five years following ACT introduction found that only 35% of febrile patients were treated according to test results [16]. Another survey revealed that the nationally recommended first-line treatment (ASP) was prescribed in only 44% of prescriptions [17]. Eight years after introduction of public policy aimed at harmonizing effective and appropriate anti-malarial treatment across the country, the patterns of ACT use in Sudan remain largely unclear.

In this study we aimed to systematically explore patterns of ACT use among health care providers in primary health care units in Sudan, and to assess the significance of the findings in the context of risks for drug resistance. We suspected that the results of this investigation could provide a useful quantitative analysis of specific ACT prescribing problems that are common in Sudan, and could potentially help to inform strategies for promoting rational use of ACT nationally.

## Methods

### Study setting

This was a cross-sectional study conducted in Gezira State, which is located in the east-central region of the Sudan. Gezira is a large state with a total area of 27,549 km [18]. Administratively, it is divided into eight localities, containing 65 hospitals and greater than 800 primary health care facilities.

### Study population

The study population consisted of patients that sought medical care at primary health care facilities throughout Gezira State and who were diagnosed with uncomplicated malaria. Uncomplicated malaria was confirmed by the demonstration of asexual forms of the parasite in the thick or thin peripheral blood smear or by rapid diagnostic test in the presence of fever. We based our sampling method and sample size calculation on guidelines published by WHO (“How to Investigate Drug Use in Health Facilities”) [19]. Patients were recruited through a three-step sampling methodology in which we took advantage of existing administrative divisions of the state and clustering at the level of primary health centers. Firstly, five of Gezira State’s 8 localities were randomly selected for participation in the study. Second, 40 “clusters” (i.e., primary health centers) within those five states were randomly selected. Finally, 30 patients were recruited from each primary health center included in the study in order to arrive at the sample size of 1,200 patients. The study took place over a 5 month period (July-November 2011). During the study period, study teams visited each primary health center and recruited the first 30 consecutive patients who were diagnosed with uncomplicated malaria and who verbally consented to be in the study. In health facilities with high patient volume, only 2–3 days were required to recruit the necessary number of patients. In other health facilities with lower patient volumes, up to 12 days were needed to recruit 30 patients diagnosed with uncomplicated malaria.

### Data collection and analyses

Data were collected prospectively. The primary outcome of this study was the proportion of patients who were adequately managed according to the nationally recommended treatment guidelines. Adequate management was defined by patient history of fever, positive blood smear for malaria, and first-line anti-malarial treatment prescribed in the correct dose.

Seven core drug use indicators drawn from WHO’s prescribing and patient-care indicators [19] were used to assess key practices of health care providers. The prescribing indicators were: anti-malarial prescribed in generic name, antibiotic co-prescribed, analgesic co-prescribed, and anti-malarial dosage form correctly written. The patient-care indicators were: anti-malarial prescribed was fully dispensed, anti-malarial adequately labeled, and patient adequately informed about the prescribed anti-malarial. We also measured an additional 5 indicators that were developed for the purpose of this study in order to evaluate essential components of the nationally recommended protocol for diagnosing and treating malaria correctly in Sudan. These supplementary indicators were: patient history of fever, whether or not a clinical examination was performed, blood smear evaluation, positive blood smear, and correct diagnosis (as defined by patient history of fever and positive blood smear).

Study staff reviewed patients’ prescriptions to collect data relating to prescribing indicators. Exit interviews with patients were conducted to explore patient-care practices. Data collectors attended an intensive training workshop prior to the start of the study to help ensure standardized data collection. Training components included familiarization with drug use indicators, how to properly extract information from anti-malarial drug-containing prescriptions, how to interview patients, and how to record and code indicators. At study sites, data collectors recruited patients at the pharmacy when patients came to collect their medicines following clinical encounters. Through a verbal consent process, data collectors explained the purpose and risks of the study to patients. Those who consented to participate were interviewed and their prescriptions were reviewed. Data were recorded on standardized forms and coded.

### Statistics

Chi-squared test was utilized to compare frequencies of prescription, patient-care, and supplementary indicators between types of anti-malarial medications prescribed. Data were analyzed using SPSS 22.0 (IMB SPSS Inc., Chicago, IL, USA).

### Ethical considerations

Ethical approvals for this study were obtained from Al Neelain University Institutional Review Board, the Sudan National Fund for Promoting Medical Services, and Gezira State Ministry of Health. Verbal-only consent was approved by the three boards, as it was not practical to obtain written informed consent from all patients.

## Results

The five localities within Gezira State that were randomly selected for this study were Greater Medani, Gezira South, Gezira East, Hasahisa, and Kamleen. The names of the 40 primary health centers within these five localities that were randomly selected for participation are being kept anonymous to maintain confidentiality of patients and health providers.

Of the 1,200 prescriptions reviewed, 88.6% included ACT (Table 1). Of these, virtually all were ASP; only two ACT prescriptions contained AL. One hundred and fourteen (114) patients were prescribed artemether injections, comprising 9.5% of the prescriptions. As the main focus of this was ACT prescribing practices, we restricted subsequent data analysis to the 1,175 patients (98%) who were prescribed either ASP or artemether injections.

**Table 1:** **Anti-malarial treatment formulations prescribed in 40 primary health centers in Gezira State, Sudan**

	**n**	**%**
Artesunate-sulfadoxine/pyrimethaminetablets	1,061	88.4
Artemether injection	114	9.5%
Quinine injection	14	1.2%
Quinine tablets	5	0.4%
Sulfadoxine/pyrimethamine tablets	4	0.3%
Artemether-lumefantrine tablets	2	0.2%
**Total**	1,200	100.0%

Just fewer than 41% percent of the 1,200 patients in the study were correctly diagnosed with uncomplicated malaria, and 26.9% of patients were adequately managed for uncomplicated malaria according to the nationally recommended treatment guidelines.

The frequencies of prescribing, patient-care, and supplementary indicators stratified by anti-malarial medicine prescribed are presented in Tables 2, 3 and 4. Only 11.8% of prescriptions contained the generic name, and strong evidence of association existed between prescribing in generic name and the type of anti-malarial prescribed (p <0.000). Although ASP was prescribed in greater than 90% of prescriptions, it was written generically in only 6.7% of prescriptions. It was observed that many prescribers tended to write the informal name for ASP (“Rajimat” which translates to “missiles” in English). Conversely, artemether, while incorrectly prescribed, was written generically in 58.5% of prescriptions. Nearly 25% of prescriptions contained an antibiotic without a clear indication for its use.

**Table 2:** **Prescribing indicators stratified by anti-malarial medication prescribed**

	**ASP n (%)**	ART* n (%)	**All prescriptions n (%)**	**95% CI**	**Chi-squared p value**
	**N = 1,061**	**N = 114**	**N = 1,175**		
Anti-malarial prescribed in generic name	71 (6.7%)	67 (58.8%)	138 (11.8%)	10-13.6	0.000
Antibiotic co-prescribed	248 (23.4%)	36 (31.6%)	284 (24.2%)	21.8-26.7	0.000
Analgesic co-prescribed	342 (32.2%)	84 (73.7%)	426 (36.3%)	33.6-39.1	0.065
Anti-malarial dosage form correctly written	769 (72.5%)	80 (70.2%)	849 (72.3%)	69.7-74.9	0.584

*Artemether injection.

**Table 3:** **Patient-care indicators stratified by anti-malarial medication prescribed**

	**ASP n (%)**	ART* n (%)	**All prescriptions n (%)**	**95% CI**	**Chi-squared p value**
	**N = 1061**	**N = 114**	**N = 1175**		
Anti-malarial prescribed was fully dispensed	1,028 (96.9%)	104 (91.2%)	1,132 (96.3%)	95.1-97.3	0.006
Anti-malarial adequately labeled	34 (3.2%)	0 (0.0%)	34 (2.9%)	2-3.9	0.069
Patient adequately informed about the prescribed anti-malarial	510 (48.1%)	64 (56.1%)	574 (48.9%)	46-51.8	0.115

*Artemether injection.

**Table 4:** **Supplementary indicators stratified by anti-malarial medication prescribed**

	**ASP n (%)**	ART* n (%)	**All prescriptions n (%)**	**95% CI**	**Chi-squared p value**
	**N = 1,061**	**N = 114**	**N = 1,175**		
Patient presented with history of fever	891 (84.0%)	90 (78.9%)	981 (83.5%)	81.4-85.6	0.184
Patient clinically examined	221 (20.8%)	28 (24.6%)	249 (21.2%)	18.9-23.5	0.338
Peripheral blood smear for malarial obtained	1,029 (97.0%)	105 (92.1%)	1,134 (96.5%)	95.5-97.6	0.013
Peripheral blood smear positive for malaria	516 (50.1%)	73 (69.5%)	589 (51.9%)	49-54.8	0.000
Patient correctly diagnosed with uncomplicated malaria	425 (40.1%)	56 (49.1%)	481 (40.9%)	38.1-43.7	0.071

*Artemether injection.

Regarding dispensing practices, the prescribed anti-malarial was fully dispensed in the vast majority of cases, signifying a high availability of anti-malarial medications at the study sites. However, labeling practices were adequate in only 2.9% of dispensed treatment packages. More than half of patients were inadequately informed about their anti-malarial treatment.

## Discussion

More than eight years have elapsed since the introduction of ACT for malaria in Sudan. However, proper case management for patients with malaria remains a challenge. In this large study of prescribing practices among healthcare workers in Gezira State, the recommended first-line drug was prescribed in most patients, which would seem to indicate confidence among heath care providers in this approach to treatment. Nevertheless, misuse of ACTs was widespread. Artemether injections were prescribed inappropriately, patients were not diagnosed according to standard guidelines, patients received inadequate education regarding their therapy, and treatment packages were poorly labeled. Overall, a minority of patients were diagnosed and treated according to the nationally recommended guidelines. A previous study in Ghana showed similar results [20].

A variety of factors may explain the findings in this study. For instance, poor supervision of health care providers, coupled with inadequate training and few opportunities for continuing education, could be a contributing cause of irrational ACT prescribing. Patient-related factors may also play a large role. Self-treatment of malaria is a common practice in Sudan [21], as well as in other countries [22]. Patient demand for treatment may influence the prescribing behaviors of health care providers. This may be especially true at the primary health care level whereworking conditions are sometimes unfavorable due to heavy workloads and low salaries. Additionally, it is not infrequent that such facilities encounter medication stock outs, which can lead by necessity to haphazard prescribing of other anti-malarial drugs (e.g., prescribing artemether injections for uncomplicated malaria despite the fact that this is not recommended therapy).

The majority of health care providers in this study requested a laboratory confirmation for malaria before prescribing treatment. However, among those who were prescribed ASP, only half were smear positive. Higher rates were reported in a Kenyan study where nearly 80% of patients with negative blood smears were prescribed malaria treatment [23]. This may raise concerns about Sudanese healthcare worker’s acceptance of laboratory results. Lack of trust of health care providers in laboratory diagnosis could be a factor in overreliance on clinical diagnosis of malaria. In the past, when laboratory facilities were largely unavailable in rural and remote areas, presumptive treatment of malaria was widely accepted. In recent years, however, with the expansion of health services across the country including the introduction of rapid diagnostic tests, presumptive treatment is no longer recommended as long as laboratory facilities are available. Moreover, requesting a laboratory investigation without utilizing its result is a waste of resources and poses unnecessary cost for patients. Most importantly, prescribing ACT for malaria negative patients increases the risk of developing drug resistance in the future, and should therefore be restricted. Unless all efforts come together to ensure accurate and safe diagnosis of malaria patients, barriers to effective clinical practices are likely to remain.

According to the national malaria treatment policy, the first-line treatment is made available free of charge in primary health care facilities. Overall, prescribing ACT using the generic name was widely neglected. Prescribers tend to use the term “Rajimat” to refer to the first–line therapy instead of prescribing it generically. It appears that the system for reviewing written prescriptions is either lacking or ineffective. Furthermore, the rate of antibiotic co-prescribing is evidently high. In some cases the healthcare provider may be uncertain about the diagnosis and therefore prescribe an antibiotic along with the anti-malarial. Haphazard antibiotic prescribing promotes the development of drug resistance and puts patients at risk of adverse drug effects. Our investigation also showed that the ACT dosage form was incorrectly written in the majority of prescriptions. For a prescription to be considered correctly written it should at minimum contain the medication’s dose (written in milligrams), quantity, and schedule. That information was incorrect, incomplete, or not written in the vast majority of prescriptions reviewed in our study. This practice has serious implications related to patient safety, including increasing the potential for treatment failure, promoting drug resistance, and increasing the risk of complications either due to the disease itself or to the administering of an inappropriate dose.

Interestingly, the availability of ACT in the study sites was high during the study period. Most ACT prescriptions were fully dispensed. This is a positive finding, since reliable availability of first-line therapies at primary health facilities would be expected to promote their rational use. However, labeling of dispensed drug packages was grossly inadequate. Moreover, information given to patients about their prescribed treatment was insufficient in most cases. Patient education and information enhance their adherence to prescribed therapies, leading to better treatment outcomes [24-26]. Heavy patient load in primary health facilities could be a main contributory factor.

Given that a multifactorial etiology is likely the cause for poor ACT prescribing patterns among health workers in Sudan, it would seem difficult to significantly improve the situation unless collaborative efforts take place by many different stakeholders. Examples of potentially important interventions include targeted training programmes for health workers, strategies for providing clear ACT information to patients and to the general public, strict policies focused on ACT deployment, and continuous monitoring of existing practices. Clear regulations relating to ACT use should be institutionalized, and more support is needed to encourage health care providers to adhere to the recommended guidelines. Evidence-based interventions such as implementing a self-administered checklist have proven to be effective for improving the performance of health care providers in disciplines such as surgery and childbirth [27,28]. Perhaps similar checklist-based interventions could promote the rational use of ACTs in Sudan.

This study has a several limitations. Prescribing data in this study were collected prospectively over a limited period of time and the fact that healthcare providers were aware that their practices were being observed could be a source of bias via the Hawthorne effect. However, retrospective collection of information was not feasible in this setting since records in most health facilities are severely incomplete. Interruptions in the anti-malarial drug supply chain or seasonality were also possible sources of bias. Another limitation of this study is the cluster sampling method used. While it is a well-accepted statistical method for increasing efficiency of data capture, similarities between individuals within clusters could result in the study sample being less representative of the study population. Nevertheless, Gezira state covers a huge geographical area and random sampling was impractical.

## Conclusion

The results of this study suggest that poor anti-malarial prescribing practices are prevalent in Sudan. This has important public health implications since failure to adhere to nationally recommended guidelines could play a role in the development of drug resistance. As such, there may be urgent need for identifying ways to improve the anti-malarial prescribing practices of heath workers in Sudan. Understanding the specific behaviors of health workers, such as those highlighted in this investigation, may help to provide a blueprint for how to tailor quality improvement interventions that will be successful.

## Abbreviations

ACT: Artemsinin-based combination therapy
AL: Artemether-lumefantrine
ART: Injectable artemisinin
ASAQ: Artesunate-amodiaquine
ASMQ: Artesunate-mefloquine
ASP: Artesunate-sulfadoxine/pyrimethamine
DHAPQ: Dihydroartemisinin-piperaquine
WHO: World Health Organization
